# Potential Applications of Some Indigenous Bacteria Isolated from Polluted Areas in the Treatment of Brewery Effluents

**DOI:** 10.1155/2018/9745198

**Published:** 2018-02-11

**Authors:** Temesgen Oljira, Diriba Muleta, Mulissa Jida

**Affiliations:** ^1^Microbiology and Microbial Biotechnology Graduate Program, Department of Biology, Wollega University, Nekemte, Ethiopia; ^2^Industrial Biotechnology Unit, Department of Biotechnology, Wolkite University, Wolkite, Ethiopia; ^3^Environmental Biotechnology Unit, Biotechnology Institute, Addis Ababa University, Addis Ababa, Ethiopia; ^4^Environmental Biotechnology Directorate, Ethiopian Biotechnology Institute, Addis Ababa, Ethiopia

## Abstract

Biological wastewater treatment is economically feasible and ecofriendly. This study was aimed at isolating bacteria from brewery wastes and evaluating their bioremediation potential as individual isolate and/or their consortium in reducing the pollutants of brewery effluents. A total of 40 bacterial isolates were recovered and of these the three best isolates were selected. The selected bacteria were identified to genus level by using morphological and biochemical characteristics. Accordingly, the isolates were identified as* Aeromonas *sp.,* Pseudomonas *sp., and* Bacillus *sp. After 12 days of incubation, the removal efficiency of these three isolates and their combinations for biological oxygen demand and chemical oxygen demand varied from 73.55% to 94.85% and 76.78% to 93.25%, respectively. Total nitrogen and phosphorus removal was within the range of 54.43% to 77.21% and 41.80% to 78.18%, respectively. Total suspended solid, total solid, and total dissolved solids removal ranged from 66.74% to 90.3%, 54.69% to 88.5%, and 53.02% to 88.2%, respectively. The pH and electrical conductivity values ranged from 6.81 to 8.65 and 3.31 mS/cm to 3.67 mS/cm, respectively. The treated effluent increased* Beta vulgaris* seeds germination from 80% to 100%, with mean germination time of 3.1 to 5.2 days and seedlings length of 2.3 cm to 6.3 cm. Therefore, the development of this finding into a large scale offers an attractive technology for brewery waste treatment.

## 1. Introduction

Industries are major source of pollution in all environments. Brewery plants have been known to cause pollution by discharging effluent into receiving stream, ground water, and soil in Ethiopia [1]. Environmental concern that can be associated with brewery wastewater include biochemical oxygen demand (BOD), chemical oxygen demand (COD), total suspended solid (TSS), pH, nutrients (nitrogen and phosphorus) concentration, and temperature [2]. Brewery industry wastewater contains organic waste with pollution levels depending on the beer production process and capacity of water consumption during the process [3].

Untreated brewery effluents typically contain suspended solids in the range 10–60 mg/l, BOD in the range 1,000–1,500 mg/l, COD in the range 1800–3000 mg/l, and nitrogen in range 30–100 mg/l [4]. Effluent pH can fluctuate from 3 to 12 depending on the use of acid and alkaline cleaning agents as well as the temperatures average becomes about 30°C [5].

Currently, in Ethiopia, there are ten breweries. It is reported that the majority of brewery industries in Ethiopia discharge their wastewaters into nearby water bodies and open land with little or no prior treatment [1]. The effluent from rural breweries is used for irrigation purposes. However, the breweries effluent in urban areas is directly discharged into rivers without any prior treatment.

One of the wastewater treatment systems which is economically feasible and environmentally sound is a biological treatment using microorganisms [6]. This system uses microbial activity to oxidize organic compounds with the aid of molecular O_2_ into CO_2_, water, and a new cell [7]. In recent years, there has been increasing interest to the use of waste stabilization ponds to treat the brewery wastewater [8]. Another highly recommended method for the effective treatment of brewery effluent is the use of constructed wetlands [9].

Brewery and other industries are blooming in Ethiopia; as a result, pollution of the environment is increasing. Thus, isolation and identification of effective microorganisms are a novel approach to minimize the current environmental problems. The objective of this study was to assess the potential of bacterial isolates in the treatment of brewery effluents and also to confirm removal of pollutants by conducting germination test on seeds of beet root* (Beta vulgaris)*.

## 2. Materials and Methods

### 2.1. Description of the Study Area

The study was conducted at Bedele Brewery Microbiology and Wastewater Treatment Laboratory. The industry was founded in 1993, which is located at 483 km south west of Addis Ababa in Buno Bedele zone, Oromia Regional State. Bedele town is located at a longitude and latitude of 8°27′N 36°21′E and with an elevation between 2,012–2,162 meters (6,601–7,093 ft) above sea level.

### 2.2. Sampling and Sample Collection

Brewery waste samples were collected from three sources. These were brewery effluent, sludge of waste, and contaminated soil with brewery effluents. One liter of effluent, two kg of sludge, and soil samples were collected aseptically using sterile glass bottles. Samples of soil, sludge, and effluent were labeled and analyzed separately. The samples were collected three times (3x) from three sources by the interval of 15 days to get different bacterial isolates. The samples were stored at 4°C until further analysis.

### 2.3. Isolation and Purification of Waste Degrading Bacteria

The samples were serially diluted in physiological saline (0.9% NaCl w/v solution) and 0.1 ml of aliquots from appropriate dilution was spread plated on nutrient agar (NA) (Hi-Media, Mumbai, India). All the plates were incubated aerobically at 30°C for 48–72 hrs. The plates were observed for bacterial growth. Morphologically different colonies were selected from plates with countable colonies and transferred to nutrient broth (NB) (Hi-media, Mumbai, India) and checked for purity by repeated streaking on NA plates. The pure isolates were designated based on their sources of isolation, from soil (SO), effluent (EF), and sludge (SL) followed by number codes. The pure cultures were preserved on NA slant at 4°C and 50% glycerol at −10°C in duplicate for further study. The day to day experimentation was carried out with cultures maintained on plates and slants. The isolates were periodically checked for purity.

### 2.4. Preparation of Inoculum for the Experiment

Inoculum was prepared following the method outlined by Krishnaswamy et al. [10]. Sterile nutrient broth (10 ml) was prepared and a loopful of 24-hour-old selected colonies of bacterial isolates on NA were inoculated separately and incubated for 48 hrs at room temperature. The bacterial cells were recovered by centrifuging at 3500 rpm for 15 min and washed. The resulting supernatant was discarded and pellet was resuspended in 0.9% NaCl solution. The optical density (OD) was adjusted to OD_0.5_ by diluting cell suspension with 0.9% NaCl at a wavelength of 660 nm and measured using spectrophotometer (Cadas 200; Germany).

After the OD of the suspension was adjusted to OD_0.5_; 0.1 ml was spread plated on nutrient agar to estimate number of viable cells per ml of suspension. The number of bacterial cells used for inoculation is presented in Table 1. Furthermore, after the OD of each isolate was adjusted, 50 ml of cell suspension was added to 250 ml of sterilized brewery wastewater and 300 ml of total volume was prepared.

### 2.5. Design of the Study and Pollutant Removal Experiment

Experiment on pollutant removal of bacterial isolates was conducted at laboratory scale batch culture in bottle under shaking at 150 rpm. The brewery wastes were sterilized by autoclaving separately. The bacterial isolates were inoculated aseptically to that of sterilized wastes in different bottles in three duplicates. The bottles were incubated at room temperature on a shaker maintained at 150 rpm. Bottles with only brewery wastes (without addition of microbes) were used as a control. In this study, three different potential bacterial isolates were selected. Selection of potential bacterial isolates was based on pollutant removal efficiency after 12th day of incubation. These three isolates EF-01(A), SL-10(C), and SO-25(D) showed the best ability to degrade the brewery wastewater and were selected to constitute their combinations (Table 1).

The three isolates that showed the best performance in removal efficiency of the brewery wastewater pollution profile were revived from their stock cultures and subcultured. A loopful of each individual isolate of 24 hrs old on NA was aseptically inoculated into sterile nutrient broth medium (10 ml) in test tubes. The inoculated test tubes were incubated for 48 hrs at 30°C. The bacterial broth cultures were adjusted at OD_0.5_. Thereafter, well-mixed cell suspension was added to sterilized brewery wastewater. The inoculated bottles containing wastewater were incubated.

In this study, pH, EC, BOD, COD, TN, TP, TSS, TS, and TDS were selected to measure pollutants removal activity of bacterial isolates. The parameters were measured before and after inoculation of potential bacterial isolates. The measurement was carried on 0, 3rd, 6th, 9th, and 12th days for all parameters. After the measurement of the selected brewery wastewater parameters, pollutants reduction capabilities of bacterial isolates (singly or in combination) were evaluated. Percentage of pollutants removal was compared against the control (without bacterial inoculation).

### 2.6. Compatibility Test of the Selected Bacterial Species

Before combination, compatibility test was performed among the three selected potential bacteria. Each of the three selected isolates was grown at room temperature and subsequently tested by the cross-streaking method at 30°C and at 37°C [11]. The isolates were inoculated as a 1.5 cm wide streak (instead of 1 cm) diametrically across duplicate nutrient agar plates. The plates were incubated overnight at either room temperature or 37°C. The selected strains were streaked singly at right angles to the original inoculum by using a wire loop (three strains per plate). The plates were incubated at 30°C and 37°C overnight, and inhibition was recorded where the indicator strains crossed the original inoculum.

### 2.7. Characterization and Identification of Selected Bacterial Isolates

Pure colonies of selected potential bacterial isolates were characterized using morphological and biochemical tests. The colony characteristics such as size, shape, color, margin, and elevation were recorded with their biochemical tests.

#### 2.7.1. Morphological and Biochemical Characterization of Bacterial Isolates

The morphological characteristics used for identification were colony size, surface (smooth, rough, granular, and papillate), color (colorless, pink, black, red, and bluish‐green), margin (entire, wavy, lobate, and filiform), elevation (flat, raised, low convex, and dome shaped), and their shape such as bacilli/rod, cocci/spherical, and spiral under a microscope. Moreover, cell arrangement (single, chain, pair, diploid, tetrad, and cluster), gram staining, and spore forming test were considered for morphological characterization of the selected isolates. The biochemical tests performed in this study were KOH, catalase, oxidase, and Oxidation Fermentation (O/F) tests.

### 2.8. Brewery Effluent Analysis

Pollutant concentration of each treatment sample was analyzed for selected pollutant parameters following standard methods [12].

#### 2.8.1. Electrical Conductivity and pH

Electrical conductivity (EC) was measured with a conductivity meter (Oyster conductivity meter). The pH of the samples was measured with a portable pH meter (Model HI9024, HANNA Instrument) [12].

#### 2.8.2. Biochemical Oxygen Demand (BOD)

BOD was determined by respirometer method by using BOD Trak II™ instrument (HACH Company, Loveland, CO, USA) [13]. Diluted sample was transferred into BOD bottle and the instrument was sealed to prevent external atmospheric pressure changes in the test bottle at 20°C for five days. The result was shown graphically in milligrams per liter (mg/l) on a liquid crystal display.

#### 2.8.3. Chemical Oxygen Demand (COD)

The chemical oxygen demand was measured by closed reflux method using strong chemical oxidant [14]. The oxidant used for this study was a mixture of potassium dichromate (KCr_2_O_7_). Finally, COD was determined by a method of colorimetric determination using HACH DR3900 spectrophotometer (HACH Company, Loveland, CO, USA). Results were displayed in mg/l COD by spectrophotometer. If the sample actual COD was higher than the range, the sample was diluted but the real results were multiplied by dilution multiple.

#### 2.8.4. Total Nitrogen (TN)

Total nitrogen was determined by using persulfate digestion method by oxidation of all nitrogenous compounds to nitrate [13]. This was done by alkaline oxidation at 105°C to convert organic and inorganic nitrogen to nitrate. Total nitrogen was determined by analyzing the nitrate in the digester. It was measured by using DR3900 spectrophotometer (HACH Company, Loveland, CO, USA) in mg/l N.

#### 2.8.5. Total Phosphorus (TP)

To measure TP vanadomolybdophosphoric acid colorimetric method was used [13]. The intensity of the yellow color was proportional to phosphate concentration. The result was read by DR3900 spectrophotometer (HACH Company, Loveland, CO, USA) in mg/l PO_4_^3−^. Conversion of total PO_4_^−3^ to total P was calculated by using molecular weight of P in PO_4_^−3^. Therefore, Total (PO_4_^−3^) (mg/l as PO_4_^−3^) × 0.3263 = Total P mg/l [15].

#### 2.8.6. Total Solids (TS), Total Suspended Solids (TSS), and Total Dissolved Solids (TDS)

Total solids, total suspended solids, and total dissolved solids were determined by gravimetric method at temperature of 103–105°C [13]. For total solids, clean crucibles were weighed using an analytical balance (Kern PFP balance IBECOR, Germany). The crucible dish was then heated for 1 hour in an oven at 103°C, allowed to cool, and then reweighed. This was recorded as the initial weight,* B*. A 50 ml of each of the samples was introduced into the crucibles and evaporated using water bath. After evaporation for 24 hrs, the crucibles and the residues in them were dried in the oven for 2 hrs at 103°C and then cooled in a desiccator and the weight taken as final weight,* A*. The total solid was estimated using the following formula:

(1) $$\begin{array}{c} TS\left(\;mg/L\;\right)=\frac{\left(A-B\right)\times 1000}{\text{Volume (mL) of sample}}. \end{array}$$

Total suspended solid (TSS) was analyzed by filtering of the sample through the preweighed filter paper. Filter paper was then dried at 103–105°C. TSS was determined by using the following formula [13]:

(2) $$\begin{array}{c} TSS\left(\;mg/L\;\right)=\frac{\left(A-B\right)\times 1000}{\text{Volume (mL) of sample}}, \end{array}$$

where *A* was the weight of filter plus dried residue and *B* was the weight of filter paper.

Total dissolved solid (TDS) was analyzed by a well-mixed sample filtered through a standard glass fiber filter, and the filtrate was evaporated to dryness in a preweighed dish (*B*) mg and dried to constant weight at 180 ± 2°C for one hour in oven and then cooled in a desiccator and weighed (*A*) mg. The increase in dish weight represents the total dissolved solids. TDS was determined by using the following formula [14]:

(3) $$\begin{array}{c} TDS\left(\;mg/L\;\right)=\frac{\left(A-B\right)\times 1000}{\text{Volume (mL) of sample}}, \end{array}$$

where *A* is weight of dried residue + dish, mg, and *B* is weight of dish, mg.

### 2.9. Germination Test

Pollutants removal was confirmed by germination test of wastewater treated with individual and combination of the bacterial isolates. For this test, seeds of beet root* (Beta vulgaris)* were bought from Buno Bedele Seed Enterprise. Seeds of beet were sterilized with 70% v/v ethanol for five minutes, followed by repeated washings using sterilized double distilled water. Sterile plastic Petri dishes were used with double layer Whatman filter paper no. 1 in two replicates from each treatment. Ten healthy treated seeds of uniform size per Petri dishes were used. Seeds were spread at equal distance in each Petri dish lined with round filter paper. Then each labeled Petri dish with seeds was irrigated with 5 ml of different treatments of wastewater and then incubated at 25 ± 2°C [16].

Different parameters like germination percentage, mean germination time (MGT), and seedling length were recorded at different time intervals of plant growth. First recording was done after 12 hrs of incubation and subsequent recordings were done at a day interval till the 6th day of incubation. The Petri dishes were rearranged at random on every one day to ensure no systematic effects due to positioning within the incubator.

#### 2.9.1. Germination (%)

Germination in each experimental set was recorded and total germination was calculated and expressed in percentage [17]:

(4) $$\begin{array}{c} Germination\%=\frac{\sum n}{N}, \end{array}$$

whereas *n* is number of seeds geminated and *N* is total number of seeds sowed.

#### 2.9.2. Mean Germination Time (MGT)

The mean germination time was calculated using the daily counts for each lot [17].

(5) $$\begin{array}{c} MGT=\frac{\sum n\cdot D}{\sum n}, \end{array}$$

where *n* is number of seeds germinated in the *i*th time (not the accumulated number, but the number correspondent to the *i*th observation),* D* is days from the beginning of the germination test, and ∑*n* is total of germinated seeds.

#### 2.9.3. Seedling Length (cm)

The shoot length was measured from the base of the primary leaf to the base of the hypocotyl and the mean shoot length was expressed in centimeter. Root length was measured from the tip of the primary root to the base of hypocotyl and mean root length was expressed in centimeter. By adding the root length and shoot length, seedling length was calculated and expressed in centimeter.

### 2.10. Data Analysis

Statistical analysis was performed using SPSS program (SPSS; Version 20.0). The data were analyzed through one-way analysis of variance (ANOVA) at 95% confidence level to compare the performance efficiency of each individual and combination treatments. Means separation was done following Duncan's test.

## 3. Results and Discussion

A total of 40 different bacterial isolates with diverse morphological characteristics were retrieved from brewery effluent, sludge, and soil contaminated with brewery wastewater. Out of 40 isolates, the best three bacteria were selected as potential organisms for waste treatment technology.

### 3.1. Morphological and Biochemical Tests of Selected Potential Bacteria

Based on the cultural characteristics, morphological tests, and biochemical tests (Table 2), these selected bacterial isolates were assigned to three genera, namely,* Aeromonas *sp. (EF-01)*, Pseudomonas *sp. (SL-10), and* Bacillus *sp. (SO-25).

### 3.2. Brewery Effluent Analysis before and after Treatment

**Figure 1:**
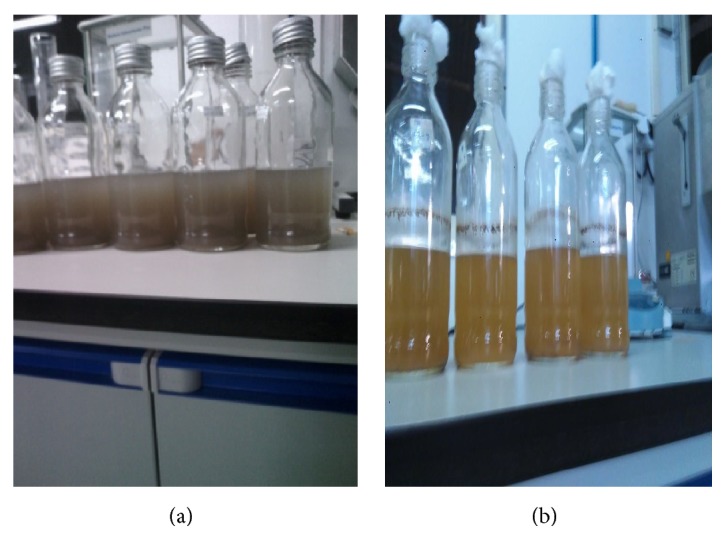
Wastewater samples before (a) and after (b) treatment.

The appearance of brewery wastes before and after treatment is shown in Figure 1. The results of the study demonstrated that after 12 days of incubation, measured parameters for pollutants decreased and their removal efficiency (%) was increased.

The pH and EC values were slightly increased. The pollutant parameter values of before and after treatment are indicated in Table 3.

Pollutant removal efficiency (%) of these three individual isolates and their combination is presented in Table 4. From individual isolates,* Bacillus* sp. had high pollutant removal efficiency for all parameters, except for TN. Similarly from combination treatments, the highest removal efficiency was in combination 2.

#### 3.2.1. Effect of Inoculation on pH and EC

**Figure 2:**
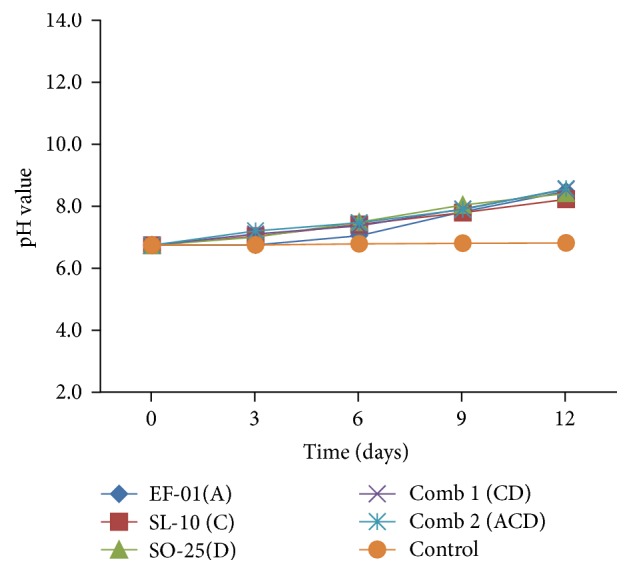
pH values of individual and combination treatments. A:* Aeromonas* sp., C:* Pseudomona *sp., D:* Bacillus* sp., and Comb: combination.

The results of the study demonstrated that pH values increased in all the treatments (Figure 2). The pH values were between 6.81 and 8.56 for all the treatments. The value of the control treatment was significantly (*p* < 0.05) lower than all the treatments. Of the individual isolates, pH value of effluent inoculated with* Pseudomonas* (SL-10) (pH = 8.22) was the lowest (*p* < 0.05) compared to all the treatments. However, the highest (*p* < 0.05) value was measured in effluent inoculated with* Aeromonas *sp. (EF-01) (pH = 8.48). The pH values of all combination treatments were significantly higher (*p* < 0.05) than all other individual isolate treatments (Table 3).

A study conducted by Choudhary et al. [18] showed that an increase of pH in treated effluent suggested that there has been activity of microorganisms that degrade organic matter. Similarly, Paramita et al. [19] have stated that degradation of proteins and organic nitrogen into ammonium (NH_4_) raises the pH and becomes alkaline. The same finding by Anggraeni et al. [20] also showed that consortium of* Cronobacter *sp. strain,* Pseudomonas fluorescens*, and* Aeromonas *sp. of bacteria isolated from soil contaminated with brewery wastewater reduced the pollutants but pH value was increased by activity of microorganisms. Gaikwad et al. [21] have also reported that* Pseudomonas* and actinomycetes reduce pollutant parameters of complex wastewater but pH value was increased.

**Figure 3:**
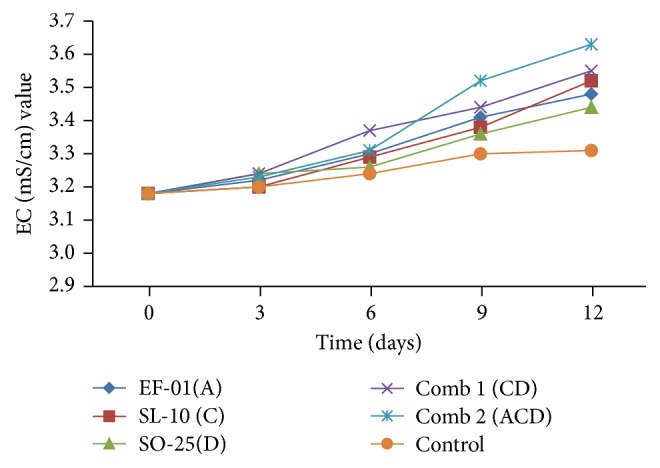
EC (mS/cm) values of individual and combination treatments. A:* Aeromonas *sp., C:* Pseudomona *sp., D:* Bacillus *sp., and Comb: combination.

Similar to pH, the EC values of all the treatments were slightly increased in all the isolates (Figure 3). EC values ranged from 3.31 to 3.67 mS/cm for all the treatments. Treatment comb 2 had high EC with value of 3.67 mS/cm with significant (*p* < 0.05) difference compared to all other treatments.

The conductivity of a solution depends on the concentration of all the ions present; the greater their concentrations, the greater the conductivity. The rise in EC values is related to the increment observed in pH values (i.e., an increase in OH^−^/H^+^ ions) that ultimately posed increase in EC values. For an acidic solution, the lower the pH is, that is, the higher the H^+^ concentration, the greater the conductivity will be. So, strongly acidic or strongly basic solution will have high conductivity [22]. In all treatments, pH values have met Ethiopian standard limit (6–9) values but none of them met for EC (1000 *μ*s/cm at 20°C).

#### 3.2.2. BOD and COD Reduction

The results of the current study showed a decline in the values of BOD and COD in the three individual isolates and their two mixed combinations (Table 3). After 12 days of incubation, all the treatments had BOD and COD values ranging from 71 mg/l to 365 mg/l and from 219 mg/l to 753 mg/l, respectively. The minimum reduction of BOD was caused by* Aeromonas* sp. (EF-01) but the maximum reduction was recorded from combined treatments (Table 3).* Bacillus *sp. (SO-25) caused considerable reduction of BOD from 1380 mg/l to 221 mg/l and COD from 3243 mg/l to 562 mg/l compared to all other bacterial isolates from day 0 to 12th day, respectively. The maximum (219 mg/l) reduction of COD was recorded in effluents inoculated with three combined bacterial isolates.

The Ethiopian standard limits for BOD and COD emission of brewery waste are 60 mg/l and 250 mg/l, respectively. From all the treatments, only samples treated with three combinations (comb 2) met the Ethiopian standard limit for COD values. In the case of BOD, none of them met the Ethiopian standard limit. However, their removal percent/efficiency was acceptable.

**Figure 4:**
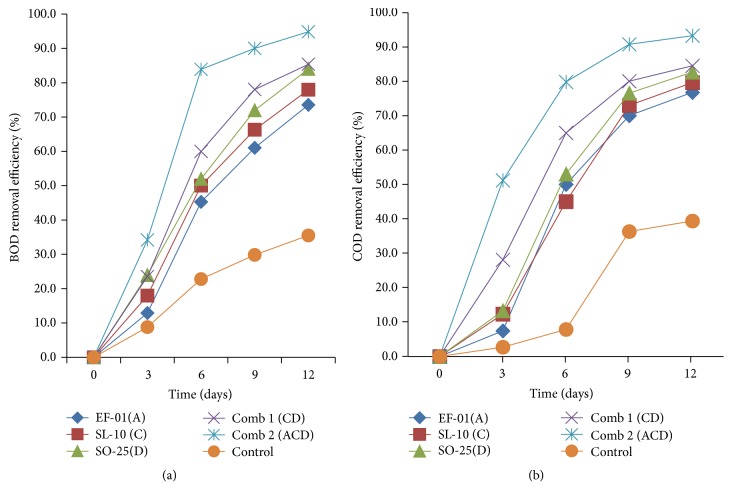
BOD (a) COD (b) removal efficiency (%) of individual and combination treatments. A:* Aeromonas *sp., C:* Pseudomona *sp., D:* Bacillus *sp., and Comb: combination.

Figures 4(a) and 4(b) show the average BOD and COD removal efficiency of each individual isolate. The removal efficiency was between 73.55% and 94.85% for BOD and 76.78% to 93.25% for COD. From individual isolates, maximum (84%) BOD removal measured in the treatment inoculated with* Bacillus *sp. but minimum (73.55%) in* Aeromonas *sp. treatment. In case of BOD removal, all the individual isolates and their combination showed statistically significant (*p* < 0.05) differences compared to the control. Likewise, tremendous COD removal was recorded by* Bacillus *sp. (82.67%),* Pseudomonas *sp. (SL-10) (79.61%), and* Aeromonas *sp. (76.78%) with significant differences (*p* < 0.05) between each individual isolates and the control.

In both BOD and COD, the maximum removal efficiency was recorded by three mixed combinations with values of 94.85% and 93.25%, respectively. The findings indicate that the reduction of BOD and COD increases in effluent inoculated with bacterial isolates with the extension of incubation period. Similarly, Metcalf and Eddy [23] have reported that the organic matter contained in the wastewater serves as a substrate for aerobic microbial metabolism which could lead to a decrease in BOD and COD concentration.

According to Hidayah and Shovitri [24], microbes are able to live based on the ability to compete for nutrients with other microbes depending on the types of nutrients present in the medium. Autochthonous microbes can adapt faster to the environment and nutrition in accordance with their origin. Microbes that are able to adapt more quickly can efficiently break down organic materials contained in the waste [25].* Pseudomonas *is a common bacterium capable of degrading pollutants [20] although not so efficient in the current study compared to other isolates. High COD reduction in the treatment of brewery effluent by* Pseudomonas *species has also been reported [26].

Further, according to Mongkolthanaruk and Dharmsthiti [27], mixed bacterial culture comprising* Pseudomonas aeruginosa*,* Bacillus *sp., and* Acinetobacter calcoaceticus *were used in the treatment of lipid-rich wastewater. Similarly, Surti [28] has reported that bacterial strains of* Pseudomonas aeruginosa, Bacillus subtilis*,* Enterobacter aerogenes, *and mixed culture of these three bacterial strains are used for COD reduction of wastewater from pharmaceutical industry.

#### 3.2.3. Total Nitrogen Removal Efficiency

**Figure 5:**
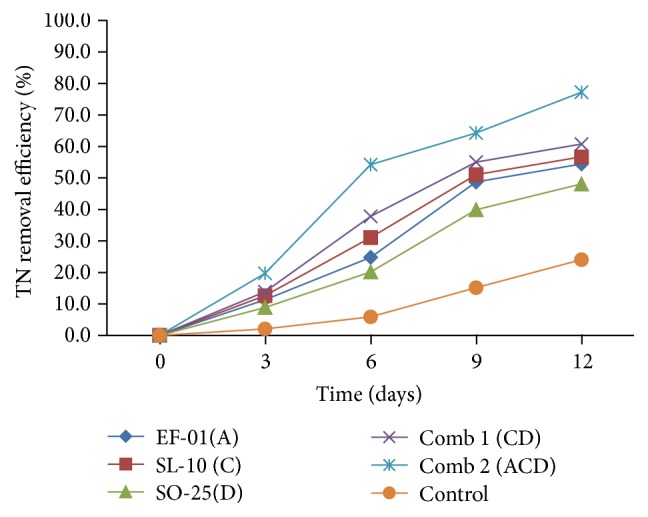
TN removal efficiency (%) of individual and combination treatments. A:* Aeromonas *sp., C:* Pseudomona *sp., D:* Bacillus *sp., and Comb: combination.

In this study, the results showed that mean TN ranged from 36 mg/l to 41 mg/l with 48.1% to 56.7% removal efficiency for individual isolates. Similarly, for combination treatment, TN values ranged from 18 mg/l to 34 mg/l with removal efficiency of 60.76% to 77.21% (Figure 5). Nitrogen removal efficiency of all the isolates and their combination treatments showed significant (*p* < 0.05) difference compared to the control (without bacterial inoculation). TN removal efficiency of the three individual isolates was also significantly (*p* < 0.05) different with each other. Out of the three isolates,* Pseudomonas *sp. (SL-10) was significantly removed TN (56.7%) followed by* Aeromonas *sp. (54.43%) and* Bacillus* sp. (48.1%).

The highest removal efficiency of TN was in the treatment with three combinations of isolates. The combination (consortium) of bacteria showed significant (*p* < 0.05) difference in efficiency of total nitrogen removal from the treated effluents compared to the individual isolates. This might be because of their synergistic effect on pollutant removal.

The Ethiopian standard limit for TN emission of brewery waste is 40 mg/l. Removal of TN for individual isolates is relatively comparable to the national effluent emission standard limit for brewery wastewater for* Aeromonas *sp. (36 mg/l) and* Pseudomonas *sp. (SL-10) (35 mg/l). In case of combination treatments both of them met the standards.

Compared to other pollutant removal parameters, TN removal efficiencies of all the isolates were lower than other parameters since most denitrifying heterotrophic bacteria are incomplete denitrifiers, which were only capable of reducing nitrates to nitrites with no further reduction of the nitrites produced. This is because incomplete denitrifying bacteria lack key nitrite reductase enzymes which enable complete denitrifiers to reduce nitrites [29]. The true denitrifying bacteria were able to reduce both nitrates as well as nitrites.* Pseudomonas *sp. is predominated among the true denitrifiers. Heterotrophic denitrifiers are common among the gram-negative bacteria such as* Pseudomonas*,* Alcaligenes*,* Paracoccus*, and* Thiobacillus*.

Similarly, among gram-positive bacteria (such as* Bacillus*), a few halophilic archaeal microorganisms (e.g.,* Haloferax denitrificans*) are able to denitrify atmospheric nitrogen under anoxic conditions [30]. In addition, the pure cultures of bacteria capable of heterotrophic nitrification that have been documented were* Alcaligenes *sp. and* Pseudomonas putida* [31], which were closely related to the autotrophic denitrifying bacteria. In this study,* Bacillus *sp*., Aeromonas *sp., and* Pseudomonas *sp. were the dominant bacterial species to involve in the denitrification processes.

Another study [32] has also confirmed that* Pseudomonas *sp. is the predominant heterotrophic bacteria involved in denitrification during activated sludge treatment. Also, Bhavan et al. [33] have reported that* Bacillus *sp. and* Pseudomonas *sp. have bioremediation ability of textile dye effluents which shows their waste degrading ability as indicated in this study.

#### 3.2.4. Total Phosphorus (TP) Removal Efficiency

**Figure 6:**
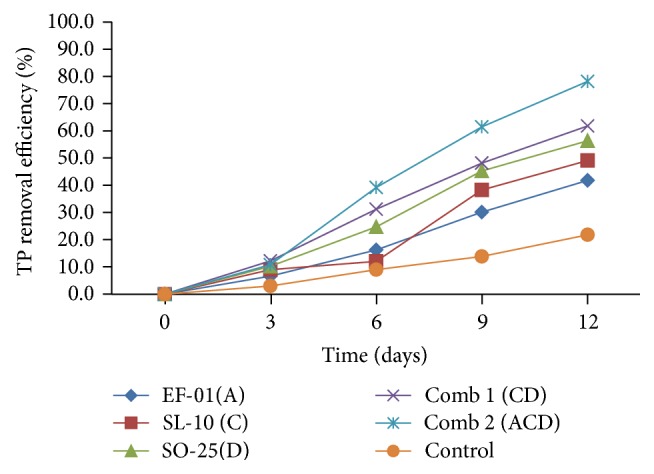
TP removal efficiency of individual and combination treatments. A:* Aeromonas *sp., C:* Pseudomona *sp., D:* Bacillus *sp., and Comb: combination.

The removal efficiency of TP is indicated in Figure 6. The TP removal efficiency of treatments inoculated with bacterial isolates was between 42.17% and 78.31%. The minimum removal was in* Aeromonas* sp. (EF-01) but the maximum, was recorded in combination 2. There were statistically significant (*p* < 0.05) differences among the bacterial isolates and the control in removal of TP.* Bacillus *sp. had higher removal than all other individual treatments with significant difference (*p* < 0.05).

From all the treatments, the highest TP removal efficiency was in combination 2 which was 78.31% with TP value of 12 mg/l. Concentrations of TP for individual isolates and their combination from the effluents did not meet national effluent emission standard limit (5 mg/l) for TP. This is because the brewery wastes contain high TP concentration (55 mg/l) before the treatment.

As in this study, Krishnaswamy et al. [10] have reported that the combination of* Bacillus *sp. and* Pseudomonas* sp. has efficiently removed the phosphate in the synthetic medium. Another study [32] has also confirmed that* Pseudomonas *sp. and* Aeromonas *sp. were capable of phosphorus accumulation as polyphosphate from activated sludge. The results reveal that among the gram-negatives, the predominant organisms in brewery wastes such as* Pseudomonas *sp. accumulate the highest PO_4_^3−^.

Study by Brodisch and Joyner [34] has suggested that gram-positive organisms such as* Streptococcus *sp.,* Micrococcus *sp*., *and* Bacillus *sp. showed reasonably high phosphate-accumulating ability. From a study conducted by Oumaima [15], pure strains of* Pseudomonas aeruginosa, Moraxella lacunata*, and* Alcaligenes denitrificans* showed remarkable efficiency in the removal of phosphate from wastewater and concluded that mixed bacterial culture strains can be used successfully for removing phosphate from wastewater.

#### 3.2.5. TSS, TS, and TDS Removal Efficiency

**Figure 7:**
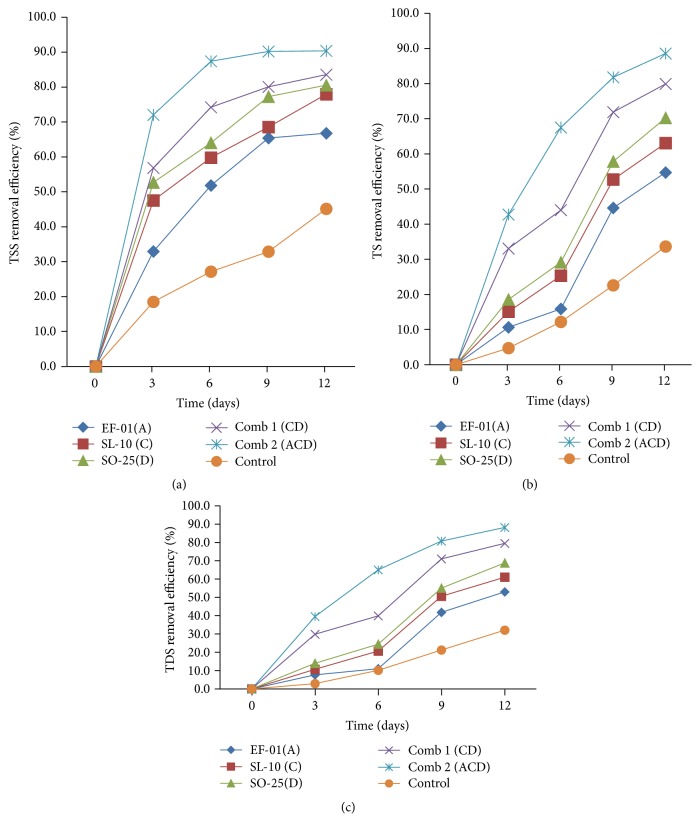
TSS (a), TS (b), and TDS (c) removal efficiency (%) of individual isolates and combination treatment. A:* Aeromonas *sp., C:* Pseudomona *sp., D:* Bacillus *sp., and Comb: combination.

The mean effluent removal efficiency of each individual treatment for TSS, TS, and TDS is presented in Figures 7(a), 7(b), and 7(c). From individual isolates, the better removal efficiency was recorded by the samples treated with* Bacillus *sp. with TSS (80.5%), TS (70.18%), and TDS (68.81%). However, the minimum removal of the pollutants was observed in uninoculated treatment with TSS of 45.1%, TS (33.64%), and TDS (32.12%). The removal efficiency of the selected bacterial isolates for TSS, TS, and TDS showed statistically significant (*p* < 0.05) differences among each other and with the control.

The result reveals that the treatment with three (3) mixed bacteria (comb 2) had the maximum TSS, TS, and TDS removal efficiency with 90.3%, 88.5%, and 88.2%, respectively. This shows that the synergistic effect of bacterial combination treatment brings about enhanced performance for effective biodegradation. As in this study, De Souza et al. [35] have reported that bacteria from different genera can work together in an environment and survive through the metabolites interaction because a mixed culture has more competence and has a higher tolerance to toxic metabolites.

*Pseudomonas* and* Bacillus* are the common known bacteria for brewery waste treatments. A study conducted by Anggraeni et al. [20] showed that* Pseudomonas fluorescens* and* Aeromonas *sp. isolated from soil contaminated with beer wastewater reduce total suspended solids (TSS) and total dissolved solids (TDS). Similarly,* Pseudomonas aeruginosa*  shows good potential for use in wastewater treatment, total suspended solids (TSS), total solids (TS), and total dissolved solids (TDS) degradation [26]. This shows waste degrading ability of* Pseudomonas *sp. as indicated in this study.

Likewise, reduction in TSS, TS, and TDS of rubber processing effluent by using* Pseudomonas* sp. has been demonstrated [36]. A study by Gaikwad et al. [21] has shown that there was a maximum reduction in TSS, TDS, TS, BOD, and COD of complex wastewater by using microbial consortia of various bacterial genera, namely,* Pseudomonas, Bacillus, Staphylococcus, *and* Streptomyces*. A study conducted by Safitri et al. [37] has shown that decrease in TSS concentration is the highest in treatment containing a consortium of* Bacillus pumilus, Bacillus subtilis, Bacillus coagulans, Pseudomonas putida, Bacillus licheniformis, *and* Nitrosomonas *sp.

### 3.3. Germination Test

Another interesting observation was that treated brewery wastes enhanced germination of beet seeds compared to the control. Mean comparison of germination percent, seedling length (cm), and mean germination time (MGT) of beet seeds are indicated in Table 5.

#### 3.3.1. Germination Percent (%)

Table 5 shows that germination percent of beet root seeds ranged from 50% to 100%. The results showed that germination percent of seed by wastewater before treatment was significantly lower than others (*p* < 0.05). The maximum germination percent was in combination treatment.

Seeds germination difference in untreated and treated wastes is due to presence of less toxic chemicals in the treated effluent compared to untreated effluent due to the presence of high level of toxic substances in the latter [38]. Similar to this finding, Pandey et al. [39] have shown that high concentration of distillery and brewery effluent have an inhibitory effect on seed germination and early growth of plants of maize and rice. Similarly, Ogunwenmo et al. [40] have reported that treated brewery effluent enhances seed germination in* Amaranthus hybridus.* Also, Yadav et al. [41] have demonstrated that sewage wastes treated with consortia of bacteria increase the percentage of germination and seedlings growth of different seeds as indicated in this study.

#### 3.3.2. Mean Germination Time (MGT)

The mean germination time (MGT) of all the treatment was 3.1 to 5.2 days (Table 5). The maximum mean germination time was in untreated brewery wastes and control which were 5.2 and 5.0 days, respectively. The minimum mean germination time was in seed germinated in wastes treated with comb 2 (3.1 days). There is an increased trend of mean germination time value of the plants with increasing effluent concentrations, because of the high amount of total solids in the effluent that disturbs the osmotic relations of the seed and retarding seed germination.

A study conducted by Orhue et al. [42] showed that treated brewery effluent decreases the mean germination time of maize. Andleeb et al. [43] have found that tannery effluents caused a reduction in growth of sunflower parameters along with other parameters like chlorophyll content, protein, carbohydrate content, and so forth. Similar observations have been reported by Manu et al. [38] as indicated in this study.

#### 3.3.3. Seedlings Length (cm)

The seedlings length of germinated beet seed after six days of incubation was minimum (2.3 cm) and maximum (6.3 cm). The minimum seedlings length was in seeds germinated in untreated wastewater, followed by control treatment. The maximum germination occurred in three combinations of isolates comb 2 (ACD), followed by comb 1 (CD). This is because untreated wastewater contains high amount of toxic substances such as high amount of total solids in the effluent; as a result germinated seed became dry [38].

In agreement with the present study, the reduction in seedling (root and shoot) lengths with the elevated amounts of total dissolved solids at higher concentrations has been demonstrated elsewhere [44, 45]. This could also be related to the fact that some of the nutrients present in the effluents are essential but at high concentration they become hazardous. Therefore, plants irrigated with treated effluents have higher germination percentage, seedling length, and lower mean germination time (MGT) as indicated in this study.

## 4. Conclusion

Based on the results obtained with the brewery wastewater biotreatment experiments,* Aeromonas *sp. (EF-01),* Pseudomonas *sp. (S L-10), and* Bacillus *sp. (SO-25) were capable of reducing the pollutant parameters such as pH, EC, BOD, COD, TN, TP, TSS, TS, and TDS from the brewery wastes.

In this study, the maximum pollutant removal occurred in brewery effluents inoculated with combined bacterial isolates for all parameters indicating their synergistic effect on degradation of wastes. The result also revealed that brewery wastes treated with* Aeromonas *sp. (EF-01),* Pseudomonas *sp. (SL-10), and* Bacillus* sp. and their mixed consortia showed enhanced germination parameters.

Generally, it can be concluded from the treatment performance of this experiment that data generated from this study can give an insight for the use of potent bacterial isolates as an alternative wastewater treatment technology.

Therefore, the development of this experimental system into a large-scale working unit offers an attractive alternative technology for waste treatment.

## Figures and Tables

**Table 1:** Treatment design and number of bacteria cells (CFU) estimated per ml of suspension.

Treatment	Isolate code	Designation	Mean colony forming unit per ml of suspension × 10^6^ CFU/ml
T1	EF-01(A)	A	2.75 ± 10
T2	SL-10(C)	C	2.85 ± 17
T3	SL-25(D)	D	2.77 ± 14
T4	Comb 1	C + D	2.76 ± 24
T5	Comb 2	A + C + D	2.76 ± 26
T6	Control	Not inoculated	—

T: treatment and Comb: combination.

**Table 2:** Morphological and biochemical characteristics of selected bacterial isolates.

Test	EF-01	SL-10	SO-25
Surface	Smooth	Smooth	Rough
Colony shape	Circular	Irregular	Irregular
Color	Greenish yellow	white	White
Margin	Entire edge	Filamentous	Lobate
Elevation	Flat	Convex	Raised
Cell shape	Rod	Rod	Rod
Gram stain	−	−	+
KOH test	+	+	−
Endospore	−	−	+
Catalase test	+	+	+
Oxidase test	+	+	+
O/F test	F^+^	O^+^	−
Suggested genus	*Aeromonas *sp.	*Pseudomonas *sp.	*Bacillus *sp.

O: oxidative, F: fermentative, +: positive, −: negative, SL: isolated from sludge, EF: isolated from effluent, and SO: isolated from soil.

**Table 3:** Effluent analysis before and after treatment for 12 days.

	Number	Treatment	Parameters								
			pH	EC mS/cm	BOD (mg/l)	COD (mg/l)	TN (mg/l)	TP (mg/l)	TSS (mg/l)	TS (mg/l)	TDS (mg/l)
BT	(1)	BT	6.74 ± 0.03^e^	3.18 ± 0.01^e^	1380 ± 7^a^	3243 ± 8^a^	79 ± 4^a^	55 ± 3^a^	466 ± 13^a^	3948 ± 21^a^	3482 ± 13^a^

After treatment	(2)	EF-01(A)	8.48 ± 0.02^bc^	3.48 ± 0.02^bc^	365 ± 5^c^	753 ± 2.67^c^	36 ± 1^cd^	32 ± 1^c^	153 ± 20^c^	1789 ± 10^c^	1636 ± 20^c^
	(3)	SL-10(C)	8.22 ± 0.08^d^	3.52 ± 0.02^b^	304 ± 6^c^	661 ± 3.67^d^	35 ± 6^d^	28 ± 3^cd^	103 ± 7^d^	1461 ± 105^d^	1357 ± 100^d^
	(4)	SO-25(D)	8.41 ± 0.1^c^	3.44 ± 0.06^c^	221 ± 12^d^	562 ± 8^e^	41 ± 5^c^	24 ± 5^de^	91 ± 5^de^	1177 ± 10^e^	1086 ± 10^e^
	(5)	Comb 1	8.54 ± 0.04^b^	3.49 ± 0.01^bc^	202 ± 5^d^	504 ± 20^f^	31 ± 3^d^	21 ± 1^e^	77 ± 2^e^	796 ± 4^f^	714 ± 10^f^
	(6)	Comb 2	8.65 ± 0.03^a^	3.67 ± 0.03^a^	71 ± 0.3^e^	219 ± 4^g^	18 ± 0.04^e^	12 ± 0.97^f^	45 ± 4^f^	456 ± 5^g^	410 ± 3^g^
	(7)	Control	6.81 ± 0.05^e^	3.31 ± 0.02^d^	890 ± 100^b^	1966 ± 10^b^	60 ± 3^b^	43 ± 1.8^b^	256 ± 6^b^	2620 ± 20^b^	2364 ± 14^b^

*Note*. Means followed by similar letters in each column are not significantly different at the 5% level (Duncan test). Comb: combination, BT: before treatment, A: *Aeromonas* sp., C: *Pseudomonas* sp., and D: *Bacillus* sp.

**Table 4:** Pollutant removal efficiency (%) of three selected isolates and their combination after 12 days of incubation.

Treatment	Parameters and their removal efficiency (%)								
	pH	EC (mS/cm)	BOD	COD	TN	TP	TSS	TS	TDS
EF-01(A)	8.48^bc^	3.48^bc^	73.55^e^	76.78^e^	54.43^c^	41.8^e^	66.74^e^	54.69^e^	53.02^e^
SL-10(C)	8.22^d^	3.52^b^	77.97^d^	79.61^d^	56.7^b^	49.1^d^	77.9^d^	63.0^d^	61.02^d^
SO-25(D)	8.41^c^	3.44^c^	84.0^c^	82.67^c^	48.1^d^	56.36^c^	80.5^c^	70.18^c^	68.81^c^
Comb 1(CD)	8.54^b^	3.49^bc^	85.36^b^	84.50^b^	60.76^b^	61.8^b^	83.5^b^	79.84^b^	79.50^b^
Comb 2(ACD)	8.65^a^	3.67^a^	94.85^a^	93.25^a^	77.21^a^	78.18^a^	90.3^a^	88.5^a^	88.2^a^
Control	6.81^e^	3.31^d^	35.52^f^	39.38^f^	24.05^e^	21.8^f^	45.1^f^	33.64^f^	32.12^f^

*Note*. Means followed by similar letters in each column are not significantly different at the 5% level (Duncan test). Legend: as listed in Table 3.

**Table 5:** Germination percent (%), mean germination time (MGT), and seedling length (cm) after 6th day.

S/N	Treatment	Germination%	MGT (days)	Seedling length (cm)
(1)	EF-01(A)	80.00^a^	4.19^bcd^	5.60^a^
(2)	SL-10(C)	80.00^a^	4.90^abc^	5.95^a^
(3)	SO-25(D)	85.00^a^	4.71^abc^	6.05^a^
(4)	Comb 1 (CD)	95.00^a^	3.50^de^	6.30^a^
(5)	Comb 2 (ACD)	100.00^a^	3.10^e^	6.25^a^
(6)	Control	55.00^b^	5.00^ab^	3.10^bc^
(7)	Before treatment	50.00^b^	5.20^a^	2.30^c^
(8)	Tap water	100.00^a^	4.10^cd^	3.80^b^

Mean values (of triplicates) followed by the same letters in each column are not significantly different (*p* > 0.05) at 95% confidence interval.
